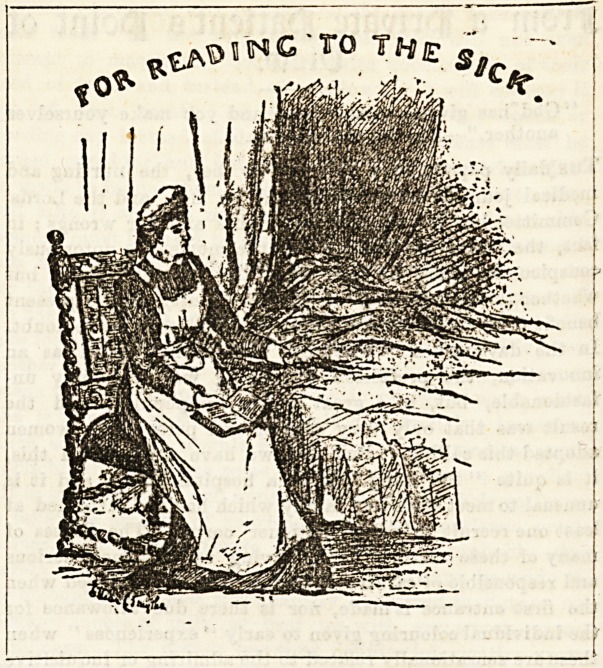# The Hospital Nursing Supplement

**Published:** 1892-10-22

**Authors:** 


### "The Hospital" Nursing Mirror.

Being the Extra Nursing Supplement of "The Hospital" Newspaper.

[Contributions for this Supplement should be addressed to the Editor, The Hospital, 140, Strand, London, W.C., and should have the word "Nursing" plainly written in left-hand top corner of the envelope.]

### En Passant.

**Tottenham Temporary Fever Hospital.**—In the notice of this new building in our number for October 8th, we mentioned the nursing arrangements as being arranged by the Superior of the All Saints' Sisterhood; it is, however, the Sister Superior of St. John's House, Norfolk Street, who, with others of the staff, has been working up these details into order, and many of the nurses of St. John's are working at Tottenham. Sister Dorothea, who is the Sister Superior of St. John's House, is also a Sister of the Community of All Saints, which fact accounts for the error.

**Bath Royal United Hospital Private Nurses.**—The "Nurses' Home" in connection with this hospital is proving an undoubted success, and where twenty-four nurses formed the staff last winter, at the beginning of this it has been found necessary to increase the number to forty-five. We learn with pleasure that the Matron's good suggestion that the nurses should receive a percentage of 5 per cent. on their earnings met with approval by the Committee and President, and the rule came into force on October 1st. At the end of three years' service an increase to 7½ per cent. will be made on the nurse's earnings. If at the end of a year it is found possible to increase the rates of percentage this will be done also. The nurses at this Home get full indoor and outdoor uniform, and the salaries at present range from £25 to £30, according to ability. The majority, however, are earning the higher rate. The rules for the staff are especially clear and just, few in number, and concise, and that in which nurses are asked to report their "slightest indispositions" to the Matron should be found in the rules of every Nurses' Home. Bath United Hospital is going forward, and we congratulate all concerned.

**The Nurses' Co-operation.** — The Nurses' Co-operation, 6, New Cavendish Street, has proved a complete and brilliant success. There are 212 nurses in the Co-operation at the present time, and the collective earnings are so large that, although only 7½ per cent. is deducted for working expenses, this has proved amply sufficient for the purpose. At the quarterly meeting of the Committee last week authority was given to pay off the money advanced by a few of the nurses as preliminary capital, and so the Co-operation has attained to a strong financial position. A word of praise is due to those nurses who, on Miss Belcher's initiative, determined to combine in order to secure for themselves a maximum return on their earnings. No doubt this speedy repayment of the capital advanced will come as a pleasant surprise, and the names of these early co-operators should be gratefully remembered for all time on the illustrious roll which contains the names of those who have made trained nursing a calling and a career for the most enlightened women. This notable success is largely due to the fact that Miss Hicks, the Lady Superintendent, has shown remarkable aptitude for the work, and her wise administration of the affairs of the Co-operation has won for it the confidence of the medical profession and of the best type of nurses. It is satisfactory to know that the accounts are audited every month by a firm of professional accountants, and that the business management is so excellent as to be worthy of imitation by all kindred institutions.

**Bolton District Nursing Association.**—The new nurses' home just opened for the nurses at Bolton seems to be a success in every way. It was originally two houses in St. George's Road, and externally the appearance remains much the same, but one has only to go inside to see that everything, through the generosity of good friends, has been done to give the nurses a really charming home. This Association started work in 1889, and its large numbers of friends give the best evidence of the usefulness of its work during the past three years.

**Anonymous Correspondents.**—There is an unfortunate number of persons in existence who think, for some reason best known to themselves, that rules and regulations are useless, aimless things, and, therefore, to be utterly disregarded. We are constantly reminding all correspondents and readers that we will insert nothing of any kind in our columns unless the contribution or correspondence is accompanied by the correct name and address of the sender, and yet we get letters from anonymous persons every week, who wish us to help them without discovering their identity. We are often also asked what our charge is for inserting letters, appointments, and notices in the columns of our paper. There is no charge at all excepting for notices which are distinctly advertisements; all we ask is that our correspondents will furnish us with evidence of good faith in the shape of name and address, and without these we can insert nothing of any kind whatsoever.

**Bangles at Bedsides.**—At the risk of being accused of harping on a familiar string we once more wish to draw the attention of all earnest workers to the fact that the "fitness" and original sense of the plain, useful, unadorned uniform does not seem to come home to many of the younger nurses as it should. Many of our readers will have noticed that bangles and trinkets generally are seen far too often on nurses in charge of a patient; we know of gold chains fastened by the tiny padlock now in vogue, which may be seen on the wrists of some of our nurses, and when we add that the other day a bangle was seen to fall from a nurse's arm into a poultice bowl, we shall convince sensible women that jewellery should find no place on a nurse's attire. Far be it from us to moralize, but is there not the danger that what is really done by want of thought may be misunderstood as want of heart? Gems and jewellery should be as absent as frills and furbelows from the dress of those who care for the sick and dying.

**Short Items.**—Nurse Cornwall and Miss Kate G. Dibb have opened a private nursing home for paying patients at St. John's Terrace, Weymouth; they will also supply trained nurses by the hour, day, or week.—Mr. Lewis Parkhurst, in last week's *Lancet*, gave particulars of the death of one more poor woman who died a victim to the unskilled and violent treatment of a midwife at Brockley.—There are now thirty-two nursing associations which have affiliated with the Q.V.J.I.N.—The Aberlour District Nurse has had a year's good work, and we are glad to hear of the undivided praise Nurse Core has met with.—Longton Sick Nursing Association has so developed that a second nurse is to be engaged.—A demonstration by the working men at Bushey Heath was admirably managed entirely by members of their own body, and they have sent £46 to the Children's Home, Caldecote House, a place the value of which they fully appreciate.

### Lectures for Asylum Attendants.

By William Harding, M.B.

V.—FOOD *(continued)*.

Such cases as are unable to masticate properly will require soft food and even spoon feeding. In any case of spoon feeding the nurse must carefully remember that one spoonful must be swallowed before another is taken into the mouth. A patient can choke on a pharynx full of semi-solid bread and milk as surely as on a chunk of beef. Especially among epileptic and paralytics there will be found patients who are approaching this condition. These should have their meat, &c., cut up for them and be kept under close observation. It is in this class that accidents most frequently happen. Suicidal and homicidal cases will of course be placed directly under the eye and within reach of the hand of the nurse who has charge of them.

Time must be given for all, even the slowest eaters, to finish. No food can be permitted to be taken away from the table. This is often a source of great trouble, as some patients will endeavour day after day to conceal bread, &c., about their person. Paralytics must be especially watched and prevented from indulging in this propensity. I have seen a death from choking occur in a paralytic who had, in passing out of the dining-room, snatched up a crust of bread unobserved, and had crammed it into his mouth unnoticed.

The forks and knives should be counted at each table separately before the patients leave their seats. Any missing article can then be localised, and at once looked for. This should never be omitted.

Epileptics.—No epileptic should ever be allowed to take ood unless under observation. The obvious danger is that the patient may have a fit and choke. When a fit does occur during a meal the patient's head should be turned to one side, and the mouth cleared so far as is possible, care being taken that a mass of food is not pushed to the back of the throat in the operation. In the later stages of epileptic dementia and in idiots, soft food and probably spoon feeding will be required. Some epileptics eat very ravenously, and do not masticate their food sufficiently. These cases will require to have their meat cut up for them or even to be supplied with crushed meat and potatoes, &c. At times it is necessary to give an epileptic her meals in a single room; on such occasions a nurse must always have the patient in sight.

Paralytics.—No paralytic should be allowed to eat alone The most dangerous period is when they are nearly reaching the soft food stage, but have not yet been put on crushed meat, &c.; they eat greedily; cram their mouths full without swallowing and will often steal anything from their neighbours' plates that they can lay their hands on. This habit a careful nurse will bear in mind. In the later stages they must be spoon-fed, and in the last of all only liquids can be taken.

The charge nurse should keep a list of those on soft food in her medicine chest, and report any patient who finds a difficulty in masticating or swallowing. This power of observing and noting changes in her cases should be encouraged. The nurse has opportunities of gaining knowledge concerning her patients' condition which the medical man has not, and she can be of the greatest assistance to him in this respect.

Patients in a state of acute excitement call for careful feeding. Their recovery is greatly a question of food and sleep, and the latter requirement is often to a great extent dependent upon the former. The continued excitement and muscular exertions require a large amount of food to repair the tissue waste and keep up the sufferer's strength. This must generally be given in a liquid form—as milk, eggs beaten up, beef-tea thickened with arrowroot, &c. It is only in some cases that the patient can be got to take a little solid food. In the very acute cases they will not even attempt to masticate. Some are quite unconscious of their need of food, and instead of swallowing it will splutter it out of their mouth, or simply blow into the spoon or feeeding-cup instead of drinking. Such patients must be nursed, cared for, and fed like babies. Frequent trials must be made to induce them to take food, as at one minute they will take it and the next will refuse it. The favourable moment must be used to get a fair amount of nourishment into the stomach, and then the nurse will endeavour to get the patient rest in the recumbent position, even if she has to lie down beside her. Sometimes these cases will take food from one person though they have refused it from another only a moment before, and that without any apparent reason for the preference. A nurse in charge of acute cases who is an adept in the administration of food is a treasure. Much anxiety and trouble is thus spared the medical man. Undoubtedly there are some persons who have a peculiar gift in this direction, and appear able to induce their patients to take any amount of nourishment. Much patience, good temper, and a sympathetic disposition, are probably the secrets of their success. A note should always be made of the amount taken, and the quantity expressed in figures, as so many ounces of milk or beef tea with arrowroot, or so many eggs. The doctor has thus definite information upon which to decide whether it is necessary to supplement this by forcible feeding. Puerperal cases will tax the nurse's powers. They are generally debilitated, and are often very excited.

A small kitchen should be attached to the ward in which acute cases are received. It is impossible to have the small amounts necessary for sick diet for special cases prepared in the general kitchen, and the present arrangement of having it made in mass is not satisfactory. The nurse could herself, with the same materials, vary their preparation, and make them more palatable. In the long run it would probably be cheaper, while the advantage to the patients would be very considerable. The monotonous dietary of our sick wards could then be varied in a way which it is impossible to attain under the present system.

*(To be continued.)*

### Christmas Competitions.

We gratefully acknowledge several kind promises of help in providing parcels of clothes for distribution among the adult patients of our London hospitals at Christmas time. In answer to correspondents the parcels must reach 140, Strand, not later than December 12th, and should be addressed to "Nursing," care of the Editor. We shall be glad if all senders will kindly write their names and addresses clearly. The prizes will be given in either books or money, as the winners choose. (1) For the best pair of socks knitted by a nurse, 5s.; (2) for the best pair of socks knitted by any Hospital reader, 5s.; (3) for the best made flannel shirt, 10s.; (4) for the best made woman's blouse, 10s.; (5) for the best made flannel petticoat, 10s.; (6) for the best made and best shaped dressing-gown for an invalid, cut out and made by nurse, 20s. It will be seen that Nos. 1 and 6 are reserved for nurses. Flannelette is cheap, and light, and warm, and would therefore form the best material for the dressing-gown. In judging, four marks are given for workmanship, four for shape, and two for general appearance; therefore, it is not wise to spend time on elaborate trimmings. Long seams may be done by machine. If our readers will ask their friends to add something to our Christmas parcels they will earn the gratitude of many a hospital worker. We shall be glad to distribute any clothing or presents of any sort besides those sent in for the competitions. To save confusion will senders kindly mark such parcels "Not for Competition."

### From a Private Patient's Point of View.

"God has given you one face, and you make yourselves another."—*Hamlet*, Act III. Sc. 1.

The daily papers have referred to her, the nursing and medical journals have discoursed on her, and the Lords' Committee has investigated her rights and her wrongs; in fact, the Nurse has become, for the moment, a notoriously conspicuous figure in the eyes of the general public, but whether the sick persons of the community derive present benefit from this state of things, is a matter open to doubt. In the days when "training" was looked upon as an innovation, the profession of nursing was not only unfashionable, but, to a great extent, unpopular, and the result was that only very earnest and persevering women adopted this calling. But now we have changed all this, it is quite "the thing" to be a hospital nurse, and it is unusual to meet with any family which has not furnished at least one recruit to the probationer corps. The fitness of many of these recruits for following up this most serious and responsible of careers, is seldom properly weighed when the first entrance is made, nor is there due allowance for the individual colouring given to early "experiences" when these are sensationally related to the admiring or inquisitive home circle.

Half the so-called "hospital scandals," and two-thirds of the unsatisfactory reputations have arisen by means of young persons, physically, mentally, or morally unsuited for ministering to the sick, and who have been carried away by a fashionable craze and a picturesque ideal of a nurse's life.

Others, again, have thought of nursing only as a means of making a livelihood for themselves, and when they devote their attention to "private" work, these are the women who frequently drive the sick man to wish for "something different" to the existing arrangements. In hospitals the order and necessary discipline tend wholesomely to efface undue prominence of any individual, but to a family, and more especially to a patient in his own home, belief in what "nurse" says becomes a kind of creed. It is only when the crisis of a dangerous illness is long past that criticism is indulged in by the sufferer, at least, this is the usual experience, although, of course, some patients are less long-suffering than others.

When a trained nurse arrives in a household, which is already disorganised by illness and its attendant troubles, she often brings with her an unnecessary amount of luggage, stored in bulky trunks, which at once causes friction with the wearied servants, and is, to say the least of it, inconsiderate. She can also annoy the family by her daintiness in demanding soda water or Burgundy, because she "cannot drink anything else," which is surely a curious position for anyone who works for a living to take up. Then the patient is perhaps entertained with a dismal history of her last case, and the details of the disease, which always sound much more dreadful than the reality in unaccustomed ears. All this may be the fruit of an ignorant or vulgar mind, quite unconscious of its own follies, but none the less is it morally harmful on that account. If, to a character such as this, an untidy appearance, a noisy chatelaine, high-heeled shoes, bangles, and rough hair, be added, we have before us a ghastly figure, and surely not a proper one to reign supreme, even temporarily, over a disordered mind or body? There are a thousand small trials in sickness, of which the healthy have small understanding, and many of these are quite needless, such as the whispers outside the chamber door, and the discussions within it, as to the silent patient's wants or wishes, whilst he lies with unstrung nerves, not realising his own power to forbid the sounds which torture him so cruelly.

Surely, if one half the world knows not how the other half lives, still less do good nurses know how bad ones work; and these latter are the section which has dulled the halo, and shattered the respect which for many years encircled and protected the true followers of Florence Nightingale.

With regard specially to dress, that test of neatness and suitability in all ranks, we find sarcastic paragraphs in leading papers, treating of the nurses' degradation to the rank of street sweepers, and in spite of this public reproof, even to-day do they return to their patients in dresses besmirched with the unsanitary contributions from London roads and pavements.

There is one special type of private nurse who might well be called "the confider," as she invariably acquaints her charges with most of her own life's history. If she has any matrimonial prospects she makes no secret of them, and she little thinks that her hearers take genuine amusement out of her little stories, and serve them up later on for the delectation of their friends and the doctor too. Speech may be silver but silence (on such subjects) is most certainly golden. Surely it was never ordained that nurses should emerge from years of training and experience to prove scourges for mankind. We do not here touch on the subject of good nurses, they, like good mothers, need praise as little as the lily does paint. We speak of "the nurse of the period" who bids fair to out-balance in numbers and in clamour the women England idolised some ten years ago. We have few idols now-a-days, and we break nearly as fast as we make them. An irreverent patient shattered one for a visitor the other day, who had charitably come to condole with his sufferings, and had somewhat injudiciously, uttered a few platitudes, "Well, madame," he said at last, "if Providence sent my illness, the Devil sent my nurse!"

### Appointments.

[It is requested that successful candidates will send a copy of their applications and testimonials, with date of election, to The Editor, The Lodge, Porchester Square, W.]

London Hospital.—Miss Kate Cooper has been appointed Sister of Victor Ward. Miss Cooper was trained at the London Hospital.

Miss Fearn, who has been trained at the Royal Infirmary, Glasgow, will shortly commence district nursing at Letham, N.B., for Lady Dempster Metcalfe's Fund.

Ashburton and Buckfastleigh Cottage Hospital.—We have great pleasure in announcing that Miss Oliver, who was trained in the Nightingale School at St. Thomas's Hospital, has been appointed Matron-Nurse at this hospital. Miss Oliver has worked at the Infirmary, Carlisle, and was for eight years at Frome Cottage Hospital.

Madras General Hospital.—Miss Hammans, of the Indian Nursing Service, has been appointed Matron of the Madras General Hospital at a monthly salary of 300 rupees. Miss Hammans was trained at the Leeds General Infirmary, where also she held successively the posts of Ward Sister and Night Superintendent.

Nursing Home, Stratford on-Avon.—We are glad to announce that Miss Annie Moseley has been elected Matron of this home. Miss Moseley trained at the West London Hospital, Hammers[illegible]; she then went as nurse to the Seamen's Hospital, Greenwich, for four years and a half, and subsequently acted as Matron *pro tem.* [illegible]o the Stratford-on-Avon Hospital and the Woolwich Cottag[illegible] Hospital.

### Nurses' Bookshelf.

### THE ART OF MASSAGE.*

The most fastidious student could scarcely desire a clearer type for his perusal than that in which "The Art of Massage," by Mrs. Creighton Hale, has been printed. This new text-book will be very welcome to Mrs. Hale's numerous pupils, to whom the book is dedicated, and it will certainly be a helpful addition to the library of many a nurse. Throughout the book numerous illustrations will be found, those showing the various positions of the hands during the different movements being especially clear.

In her short introduction, Mrs. Creighton Hale very wisely draws the attention of her readers to the fact that none of them need expect to learn massage from books, however clearly written, and that nothing but persevering practice on a living subject will achieve success in the art; the main object of the author has been to produce a simple text-book of readable bulk, containing what details of physiology, anatomy, and general information she considers necessary for an intelligent exercise of massage. In this she has succeeded.

The first chapter consists mainly of the various effects of scientific massage, and the "reason why" it has become such a valuable therapeutic agent, and in the next chapter the qualifications by the successful masseuse are given, the temperature of the operator's hands, for instance, which depends much, of course, on her state of health, is an important point which cannot possibly be overlooked. She must possess that inestimable silent tongue so valuable in all who minister to the sick; she must have a certain knowledge of anatomy and physiology, while punctuality, patience, thoroughness, and strict cleanliness in every detail are some of the other virtues which we may expect in the thorough masseuse. We next come to a simple anatomy, and following this we are given the various instruments and medicaments used in the art, and the best sort of bed on which to place a patient. A separate chapter describes the various manipulations, and each is accompanied by an illustration, while another chapter is given to the structure of muscles. Later on the structure and massage of the different limbs are described and the functions and treatment of the different organs. The nervous system necessarily fills the longest chapter in the book, and those illnesses so common in these days and so hard to bear, anæmia, neurasthenia, and hysteria, receive the attention they deserve; numerous cases of complete recovery from hysteria due to a thorough course of what has become known as the "Weir-Mitchell" treatment are cited.

A complete week's dietary and treatment of a patient, according to Dr. Weir-Mitchell's system, are given in chapter xviii from Mrs. Hale's own note book. In advocating temperate massage for children, the authoress makes some sensible remarks in the last chapter but one on the importance of judicious dietary for children, which we hope may ere long be acted upon a great deal more fully in future years than they have been in the past. Any ordinarily intelligent careful mother can, if she will, select the various foods required by nature to build up a sound, bony, muscular framework for her child; outer adornment is the greatest bar to some children's progress, their mothers' attention being concentrated on that to the exclusion of other vital matters. The same chapter contains a few remarks on Faradism, and a description of the Faradic Battery, which Mrs. Creighton Hale recommends as small, portable, convenient, and quite powerful enough in the generality of cases. In conclusion, the authoress expresses her opinion that "regular massage in advancing age prolongs life."

* "The Art of Massage," by A. Creighton Hale. Profusely illustrated. London: The Scientific Press, Limited, 140, Strand, W.C.)

### GROWTH.

The truest sign of life in an animal or vegetable is growth, and this growth is the effect of light and warmth. Men become stunted without a proper amount of air and sunshine, while a plant kept in the dark spindles up and finally withers away. Strength and beauty come from light and heat alone. As in the natural world, so in the spiritual life we must grow in grace if we would keep our souls alive, for there is no standing still. We forget this often in sickness, and let our bodily pains fill all our thoughts, and crowd out any care for holy things, till our souls become weak and lifeless. Now, if we did but know it we are robbing ourselves of all the comfort our poor weak suffering bodies so earnestly long for. We are turning from the Sun of Righteousness who shines alike upon the evil and the good, and are groping about miserably, while all the time what we cannot find is within our grasp, only our selfish love veils it from our eyes.

If we were to refuse food and nourishment for our sick bodies, should we not be looked upon as very foolish people Are we not, then, equally simple to starve our souls by turning away from the light and prevent their growing healthy and strong to bear the ills of our weak flesh?

Let us feed, then, on the Bread of Heaven, which came into the world to revive our wasting souls, for in Him, the Lord of Life, can we find everything we want. He is the true Light, which lighteneth every man that is born, only, unfortunately, like the Jews of old, we will not receive Him, and so we lose the blessings which might be ours. If, instead of moaning and bewailing our fate we will pray to God that we may see and feel this light and warmth within and around us, He will quickly shine upon us, and we shall begin to show the signs of life by growing.

Growing in what? In patience, in holiness, in submission to God's will, in contentment with whatever lot it may please our Heavenly Father to give us. And growing thus, our happiness will increase, for the warmth of our Saviour's love will fill us with love and joy and peace in believing. So let us bask in this heavenly light of "a cloudless Sun, which softly shines" and ripens us for His eternal glory. We shall continue to grow and ripen on earth until the hour when the Lord of Life will gather us into His harvest. O, blessed lot, when all our offences shall be purged away, and we, like fruitful ears, shall be gathered into God's garner for evermore.

"O praise the heavenly Sower,
Who gave the fruitful seed,
And watched and watered duly,
And ripened for our need."

### Everybody's Opinion.

*[Correspondence on all subjects is invited, but we cannot in any way be responsible for the opinions expressed by our correspondents. No communications can be entertained if the name and address of the correspondent is not given, or unless one side of the paper only be written on.]*

### DEGREES OF TRAINING.

"A Trained Nurse" writes: If the term "trained" should only be adopted by nurses who have earned a two or three years' certificate in a general hospital, what title should the nurses adopt who are sent by nursing institutions to be "trained" for one year at a general hospital in order that they may be sent out as "private nurses"? May I ask if someone will answer me in the pages of The Hospital?

### METROPOLITAN DISTRICT SCHOOLS.

"Sister Dora" writes: May I be allowed to suggest to those who are interested in the welfare of the children of the poor that something be done to improve the sanitary arrangements and comforts of the sick in our Metropolitan District Schools? It seems evident that the Board of Guardians is anxious to get the management of such places, and especially of the sick wards, into the hands of well-educated and trained ladies; but can such be expected to undertake the work with the arrangements continuing to be as they are, and, may I add, when such small salaries are offered? for this must necessarily be a consideration with many. To quote only one of several instances of bad arrangement which have come under my own observation. In one institution one very moderate-sized bath-room is made use of in the following ways: A large number of children are washed in it every day; sick children are bathed in it; all the hair-cutting is attended to there; in it all shoes and boots are blacked; all knives and forks cleaned; soiled clothing and linen collected; three pails are kept in one corner to receive rubbish, refuse of food, and soiled dressings; a gas jet is in another corner, over which the nurse's breakfast and supper is cooked; and in addition to all this in the same small room about thirty or forty dressings are done every morning, and many again in the evening, consisting chiefly of scrofulous wounds of every description, bad eyes, ears, heads, &c. The unwholesomeness of such an atmosphere can easily be imagined, and to expose open wounds in such a place seems anything but right. But until better places are fitted up for this purpose one must conclude that things will continue as they are.

### SIGNING AGREEMENTS.

"Doubtful" writes: I saw in the Hospital a few weeks ago, that a certain institution forced a nurse wishing to join the staff to sign a paper to the effect that "should the nurse leave that institution she would never take a case on her own account within thirty miles round"; and from the way that nurse writes she was not very well treated by this said institution. I know of a case in which a nurse, a native of the place, joined such an institute, and signed such a paper. She left of her own accord, and was leaving the town. The doctors in that and the neighbouring towns asked her to take the opening that was offered as a private nurse on her own account, but the nurse felt in honour bound by the paper she signed not to listen to the suggestion. She wrote, however, to the Lady Superintendent, asking for a testimonial "drawn from reports brought back from her cases," to help her to another post, but she received no answer. She then wrote to the Secretary, and after a long delay an answer came from the President, stating that such a nurse was employed by the institute from March, 1890, to May, 1891, and, it was believed, gave satisfaction, but the President could not speak from any personal knowledge, and the Lady Superintendent under whom the nurse worked was abroad. During the fourteen months that nurse was employed by the institute they had four Superintendents in succession, the one mentioned as being abroad, and under whom the nurse had worked, was only there three months out of the fourteen, and it was only during that time that the Home was really a home; during the rest of the fourteen months it was a trial to look forward to returning there after a hard case. This nurse refused a good opening of work among her own people and the doctors she knew so well and had worked for, because she felt in honour bound to the institution. Yet she did not even get the help of a good testimonial which would have enabled her to get another post elsewhere. Will anyone of the nursing world kindly answer this question for me? Is this nurse in honour bound to keep her part of the signed contract, seeing that the staff has acted so dishonourably towards her?

### ROYAL COMMISSION CHICAGO EXHIBITION, 1893.

Miss Josephine L. de Pledge, Matron, Chelsea Infirmary, Cale Street, S.W., writes: With a view to rendering the exhibits in connection with nursing as complete as possible at the forthcoming World's Fair at Chicago, I should be greatly obliged if you would permit me, on behalf of our sub-committee, to invite through the medium of your columns, the heads of all Hospital and Infirmary Training Schools for Nurses throughout the United Kingdom—whose standard of training is not less than three years—to kindly assist in the matter, by sending me, on or before December 1st, a copy of the certificate they issue, and lend any badge or medal peculiar to their institution, or which may have been obtained for any special service rendered to the cause of nursing. The committee will hold itself directly responsible for the insurance and safety of all such articles, which will be returned to the owners when the exhibition closes.

### "WANTED, A RESIDENT YARDSMAN!"

We have received the following letter from "Tyke,' and possibly some of our readers may be able to advise him: "I am an attendant in the imbecile and epileptic wards at a workhouse, and I wish to know if I am a competent man to apply for a position as boys' industrial trainer and drillmaster. I have had little experience in these kind of institutions, as this is my first place, and I have not held this many months. I am single, 33 years of age, and of good physique, and previous to this appointment I was in railway and hotel service. I have no trade, and, of course, I do not know what the duties are of the position in question, which is the cause of my writing you to see if I should have any chance, supposing I was to apply for such a position. I lately saw an advertisement in the newspapers, which ran somewhat as follows: 'Wanted, a resident yardsman, who will be required to act as boys' industrial trainer and drillmaster. Among other duties will be that of cleaning windows and other parts of the premises, taking the boys to and from the Board schools, bathing the boys, taking charge of the dormitories, and act as drillmaster.' I know nothing about drill, but I suppose it will be a very simple drill, and, of course, any sensible man could perform the other duties named. But perhaps the man would be required to have a trade and teach the boys his trade if possible, consequently I should not be a competent person to apply for such a position. I have taken The Hospital for a few weeks, and continue to do so. Hoping you will kindly inform me through 'Notes and Queries' if I should be qualified for the position of boys' industrial trainer and drillmaster. Please say 'Yes' or 'No.'"

### Presentations.

On the 1st inst. Miss White, District Superintendent of the Glasgow Sick Poor and Private Nursing Association, was, on the occasion of her leaving Glasgow, presented with a very handsome toilet dressing-case as a token of esteem from the nurses of the Association. Miss White was three and a half years District Superintendent in Glasgow, and leaves many friends behind her in the work.

The people of Brighouse have presented Nurses Nied, Waterhouse, Buckton, and Bodham, from the Bradford Nurses' Institution, with gold brooches as a testimony of gratitnde for the hard work the nurses did under exceptionally trying circumstances.

### The Result of a Chill.

### III.—SOME NOTABLE PICTURES.

Arthur Lancaster read the letter with an expression of pleasure and surprise. As he looked at the neat character, his mind, made hazy by sore illness, recalled a picture lying at the shop of Francis Lowther, and at the recollection his eyes sought the letters on the table. In a state of extraordinary brain-whirl, one evening, he remembered glancing at a note from the picture dealer. He had utterly forgotten the incident. Reaching out, he picked up a budget of letters, and, on searching, found one in the cramped handwriting of old Mr. Lowther. The date so startled him that he looked at the nurse, and said:

"Have I been delirious?"

"Slightly," she answered.

"Then that accounts for it," he said, leaning back on the pillow.

"Please untie that parcel," he said, presently.

The nurse removed the paper, and revealed a small water-colour sketch of mist-crowned hills, a dark valley, and a foreground of rocks and rushing water fringed with yellow fern.

"Stand it on the mantelpiece," he said. "Now please give me a pencil and paper," he added, eagerly.

While the nurse moved to comply with his request, the critic's gaze was levelled at the sketch, while animation gleamed in his large dark eyes. He seemed to have awakened suddenly from coma, and remembrance of the autumn holiday which had resulted in his present indisposition was as the drowsy efforts to recall a strange dream. But confused meditation gradually resolved itself to a distinct presentment of a comely, thoughtful girl's visage. It was the face that so often figured in his sick dreams—the face of Sybil Ruthven, his charming hostess at the old house on the skirt of Dartmoor. He smiled at the thought of her implicit belief in "the principal critic of the new school," though it had pleased and flattered him to come so unexpectedly upon an enthusiastic disciple in the wilderness. Was it fair play to conceal his identity? Well, in any case she should now know who he was. He sat up, and resting a writing case on his knees, wrote a four-paged answer to her note. When he had finished the letter, he asked for his cheque-book, and filled in one of the slips.

"Let this be posted at once," he said, sinking back, for the trifling exertion had been tiring to his relaxed muscles.

In a few days the convalescent was permitted to take moderate walking exercise at noon. It was a keen satisfaction to him to walk leisurely to the Palette Club, the temple of the new cult, and hear how the world of art progressed. But his first stroll was to the Arcade Gallery, where, in one of the small rooms, he found a landscape painting that afforded him extreme pleasure. Sitting down on a plush-covered settee, he scanned it with close interest, and, with folded arms, fell into a reverie. People came and went as he sat, and though a few glanced at the picture and turned to the catalogue, most passed it by almost unnoticed. Yet for Arthur Lancester, the well-known and rising art critic, that rather ordinary landscape had large and peculiar fascinations. He came again another time, and, while he stood before it, two girls, with short hair and odd faded gowns, stopped to criticise the execution.

"Very raw," remarked one. "Look at the colour of the clouds."

"Impressionism with a vengeance," said her friend.

"Sybil Ruthven, it is signed. Who is she?"

"I never heard of her."

"Nor I. It's sold, too. Well, if that sort of work is hung on the line in this exhibition the judges are madder than I thought them."

"They are emerald green, not madder," observed the other, and they walked away laughing.

Arthur Lancaster's lip curled slightly as he muttered "prigs," under his breath. If a man had sneered in that way he thought he might have given him some caustic comment on his judgment. It was not a picture of the superlative order of merit; that he was willing to admit, but it had some excellent, conscientious work in it. He set the giggling girls down for insignificant daubers, whose garb and would be familiarity with art could not disguise their innate Philistinism. He knew that a word from him would have made several collectors eager bidders for the painting. It was perhaps a pity he had not belauded it in the presence of Mr. Clare, the Irish millionaire. But the temptation of acquiring it had been too powerful, and he was selfishly proud to think it was his. It would be a permanent association with the woman who had interested him deeply, and whose image had been the central object in the visions of his sick-bed.

*(To be continued.)*

### Notes and Queries.

### Queries.

(10) *Plague of Fleas.*—What can be done to get rid of a plague of fleas in an attic? Free use of carbolic soap and powder is of little or no use. For how long would disinfection by sulphur make the room uninhabitable?—*Stella.*

(11) *Books on Nursing and Morphia and its Effects.*—Will some of your readers kindly tell me of a good work, not expensive, which would embrace all branches of nursing, and not only give treatment of cases, but explain why such is given? I also wish to hear of a book on morphia and its effects, given hypodermically and otherwise—*.E.C.*

(12) *Book on Hospital Sisters and Their Duties.*—Will you kindly recommend me a book on Hospital Sisters and their duties?—*G. B.*

(13) *Book on Monthly Nursing.*—Will you kindly recommend a book on monthly nursing?—*A. H.*

(14) *Where Can a Male Nurse Train?*—Would any of your readers tell me what hospitals take male probationers?—*H. L.*

(15) *Meath Home for Epileptics.*—Kindly give me the address of the Meath Home for Epileptics.—*P.W.K.*

### Answers.

(5) *Eastbourne* (*Nurse B.*)—The Home of Rest in Burlington Place will, we hear, take in nurses at the moderate sum of 7s. a week, and 12s. a week for a separate bedroom. The Convalescent Home at Meads, kindly mentioned by a correspondent, is not for those in good health, but for those who are recovering from illness of one sort and another.

(9) *Home for Children, Bournemouth* (*Trixey*).—Write to the National Sanatorium—offices, 28, King Street, St. James' Square—and see if they can help you. Also apply to the Herbert Convalescent Home if the child is over ten; or to St. Joseph's Home, Branksome Wood Road, where children under twelve are taken for 6s. a week.

(10) *Plague of Fleas* (*S.S.*).—We have never found anything to beat Calvert's carbolic for vermin of any sort. You must strip the paper from the walls and carbolic any cracks in the wainscoating. Keating's powder dusted into all cracks is very useful. If you will patiently scrape out with the invaluable hat pin crevices between boards and wash everything for two or three days with carbolic, you will soon lose your unpleasant visitors, but it must be done thoroughly. To disinfect with sulphur, a quarter of a pound of sulphur and a tablespoonful of turpentine must be burnt on an iron pan. You must close all openings by pasting paper, &c., and expose to fumes for at least six hours. The smell is very unpleasant for a couple of days, but free ventilation soon gets rid of it; a faint smell may be perceptible for some time.

(11) *Books on Nursing and Morphia* (*E.C.*).—For good all-round nursing books, Lewis' "Theory and practice of Medicine," or Humphrey's "Manuel of Nursing, Medical and Surgical" are as satisfactory as any. Perhaps some of our readers will suggest a book on morphia and its effects, suitable to a nurse.

(12) *Book on Hospital Sisters and Their Duties* (*G. B.*)—You cannot do better than get Miss Lückes' "Hospital Sisters and Their Duties," published by Churchill.

(13) *Book on Monthly Nursing* (*A. H.*)—A short manual for monthly nurses by Cullingworth, price 1s. 6d., published by Churchill, would probably be what you require.

(14) *Where Can a Male Nurse Train?* (*H. L.*)—Unfortunately there is great lack of training in the general hospitals for male nurses. Apply to the Hamilton Association, Park Street. See article on this subject in next week's "Mirror."

(15) *Meath Home for Epileptics* (*P.W.K.*).—This home is at Godalming, and a letter to the Secretary will ensure full particulars being sent you.

### Wants and Workers.

*Lady Superintendent, Hospital, King's Lynn* will be glad of coloured pictures (not Christmas cards) for screens for wards.

There is a pressing need of old linen, or, indeed, clean rags of any kind, linen, calico, or flannel, among the workers of the District Nursing Home at Ancoats. Parcels gratefully received by *Olga Hertz, Hon. Sec., Ardwick and Ancoats District Nurses' Home, Ardwick Green.*

## Figures and Tables

**Figure f1:**